# Presenilin 1 deficiency impairs Aβ42-to-Aβ40- and angiotensin-converting activities of ACE

**DOI:** 10.3389/fnagi.2023.1098034

**Published:** 2023-02-17

**Authors:** Yuan Gao, Yang Sun, Sadequl Islam, Tomohisa Nakamura, Taisuke Tomita, Kun Zou, Makoto Michikawa

**Affiliations:** ^1^Department of Biochemistry, Graduate School of Medical Sciences, Nagoya City University, Nagoya, Japan; ^2^Laboratory of Neuropathology and Neuroscience, Faculty of Pharmaceutical Sciences, University of Tokyo, Bunkyo, Japan

**Keywords:** Alzheimer’s disease, angiotensin-converting enzyme, amyloid β-protein, presenilin 1, familial AD

## Abstract

**Introduction:**

Alzheimer’s disease (AD) is associated with amyloid β-protein 1-42 (Aβ42) accumulation in the brain. Aβ42 and Aβ40 are the major two species generated from amyloid precursor protein. We found that angiotensin-converting enzyme (ACE) converts neurotoxic Aβ42 to neuroprotective Aβ40 in an ACE domain– and glycosylation-dependent manner. Presenilin 1 (PS1) mutations account for most of cases of familial AD and lead to an increased Aβ42/40 ratio. However, the mechanism by which *PSEN1* mutations induce a higher Aβ42/40 ratio is unclear.

**Methods:**

We over expressed human ACE in mouse wild-type and PS1-deficient fibroblasts. The purified ACE protein was used to analysis the Aβ42-to-Aβ40- and angiotensin-converting activities. The distribution of ACE was determined by Immunofluorescence staining.

**Result:**

We found that ACE purified from PS1-deficient fibroblasts exhibited altered glycosylation and significantly reduced Aβ42-to-Aβ40- and angiotensin-converting activities compared with ACE from wild-type fibroblasts. Overexpression of wild-type PS1 in PS1-deficient fibroblasts restored the Aβ42-to-Aβ40- and angiotensin-converting activities of ACE. Interestingly, PS1 mutants completely restored the angiotensin-converting activity in PS1-deficient fibroblasts, but some PS1 mutants did not restore the Aβ42-to-Aβ40-converting activity. We also found that the glycosylation of ACE in adult mouse brain differed from that of embryonic brain and that the Aβ42-to-Aβ40-converting activity in adult mouse brain was lower than that in embryonic brain.

**Conclusion:**

PS1 deficiency altered ACE glycosylation and impaired its Aβ42-to-Aβ40- and angiotensin-converting activities. Our findings suggest that PS1 deficiency and *PSEN1* mutations increase the Aβ42/40 ratio by reducing the Aβ42-to-Aβ40-converting activity of ACE.

## Introduction

Alzheimer’s disease (AD) is a degenerative disease of the central nervous system characterized by amyloid β-protein (Aβ) accumulation, intraneuronal neurofibrillary tangles, and neuronal loss (Goedert and Spillantini, 2006; Selkoe, 2011; Selkoe and Hardy, 2016). Aβ is produced by the hydrolysis of a type I transmembrane protein, amyloid precursor protein (APP), mediated by β-and γ-secretase (Haass et al., 1993; Kimberly and Wolfe, 2003). The most abundant form of Aβ is Aβ40 (containing 40 amino acids), which comprises 90% of all secreted Aβ (Selkoe and Hardy, 2016). Aβ40 exerts neuroprotective effects, functions as an antioxidant against metal-induced oxidative damage, and inhibits Aβ42 toxicity and Aβ42 accumulation in the brain (Zou et al., 2002, 2003; Kim et al., 2007; Kuperstein et al., 2010). In contrast, Aβ42 is more prone to aggregate and exhibit toxicity than Aβ40, and it is essential for amyloid deposition in the brain (McGowan et al., 2005; Selkoe and Hardy, 2016). Abnormal accumulation of Aβ42 in the brain is considered the cause of neurodegeneration and cognitive decline in AD patients (Selkoe and Hardy, 2016).

γ-Secretase, an aspartyl intramembrane protease complex that catalyzes the proteolysis of type I membrane proteins (De Strooper et al., 2012), is composed of four subunits, presenilin 1 (*PSEN1*, PS1) or presenilin 2 (*PSEN2*, PS2), Pen-2, Aph-1, and nicastrin (NCT) (Kimberly et al., 2003; Bai et al., 2015). PS1 and PS2 constitute the catalytic subunit of γ-secretase. Most *PSEN* FAD mutations are situated within or flanking the conserved hydrophobic TMDs and are missense mutations resulting in single amino acid changes, such as PS1L166P and PS1G384A (Wanngren et al., 2014). In addition, an AD-associated mutation within the PS1 gene deletes exon 9 (PS1Δexon9) due to a splicing error and results in the accumulation of the uncleaved full-length protein (Steiner et al., 1999). Mutations in *PSEN1* and *PSEN2* account for most cases of early onset familial AD (FAD) and are thought to affect Aβ generation by changing the cleavage site of γ-secretase, thereby increasing the amount of Aβ42 relative to Aβ40, which in turn triggers FAD (Bentahir et al., 2006; Kuperstein et al., 2010). However, the mechanism underlying the increase in the Aβ42/40 ratio associated with *PSEN* mutations is unclear. In addition to serving as the catalytic subunit of the γ-secretase complex, PS also has other functions. Previous studies showed that PS, especially PS1, also plays roles in protein trafficking and the maturation and cellular localization of NCT, APP, TrkB, N-cadherin, neurotrophin receptor–like death domain protein, epidermal growth factor receptor and integrin β1 (Naruse et al., 1998; Leem et al., 2002; Herreman et al., 2003; Uemura et al., 2003; Gowrishankar et al., 2004; Zou et al., 2008). In the absence of PS1 and PS2, maturation and cell-surface delivery of NCT are completely inhibited, whereas the maturation and cell-surface delivery of integrin β1 are enhanced, suggesting that PS regulates protein maturation in a bidirectional manner (Zou et al., 2008).

We previously reported that angiotensin-converting enzyme (ACE) converts toxic Aβ42 to neuroprotective Aβ40 and reduces the Aβ42/40 ratio (Zou et al., 2007; Zou and Michikawa, 2008). Inhibition of ACE or heterozygous ACE deletion significantly enhances Aβ42 deposition and increases Aβ42/40 ratio in the brain of AD model mice. ACE inhibitors are widely used clinically for the treatment of hypertension, however, compared with non–ACE inhibitor antihypertensive medications, ACE inhibitors can reduce IQ in male hypertensive patients (Liu et al., 2019). ACE plays a central role in blood pressure regulation *via* the renin-angiotensin-aldosterone system (Turner and Hooper, 2002). Somatic ACE consists of two homologous catalytic domains, the C-domain and the N-domain (Hooper and Turner, 2003). Although these domains are highly homologous, they have distinct physiological functions (Fuchs et al., 2008; Zou et al., 2009). Interestingly, only the N-domain of ACE exhibits Aβ42-to-Aβ40-converting activity, whereas the C-domain primarily exhibits angiotensin-converting activity. Notably, both of these activities are impaired after de-glycosylation of the *N*-glycan (Zou et al., 2007, 2009). The earliest-deposited Aβ species, Aβ43, can be converted to Aβ41, and this activity requires both active domains of ACE. Inhibition of ACE *via* treatment with the ACE inhibitor captopril leads to a significant increase in Aβ43 deposition in mouse brain (Zou et al., 2013). In addition, successive catalysis by ACE and ACE2 convert Aβ43 to Aβ40 (Liu et al., 2014).

Most *PSEN1* mutations found in FAD induce an increase in the Aβ42/40 ratio, but the underlying mechanism is unclear (Selkoe and Hardy, 2016). Thus, we hypothesized that the increase in the Aβ42/40 ratio associated with *PSEN1* mutations results from impairment of the Aβ42-to-Aβ40-converting activity of ACE. Here, we examined the effects of PS1 deficiency and *PSEN1* mutations on the maturation and glycosylation of ACE protein and its Aβ42-to-Aβ40-converting and angiotensin-converting activities. We found that ACE protein purified from PS1-knockout (PS1-KO) fibroblasts shows altered glycosylation and markedly impaired Aβ42-to-Aβ40-and angiotensin-converting activities. Transfection of wild-type (WT) PS1 restored these activities in PS1-KO cells; however, some PS mutants could not restore the Aβ42-to-Aβ40-converting activity of ACE in PS1-KO cells. These findings provide a novel mechanism underlying *PSEN1* mutations regulate Aβ42/40 ratio through ACE.

## Results

### PS1 deficiency altered ACE glycosylation and impaired its Aβ42-to-Aβ40-converting activity

**Figure 1:**
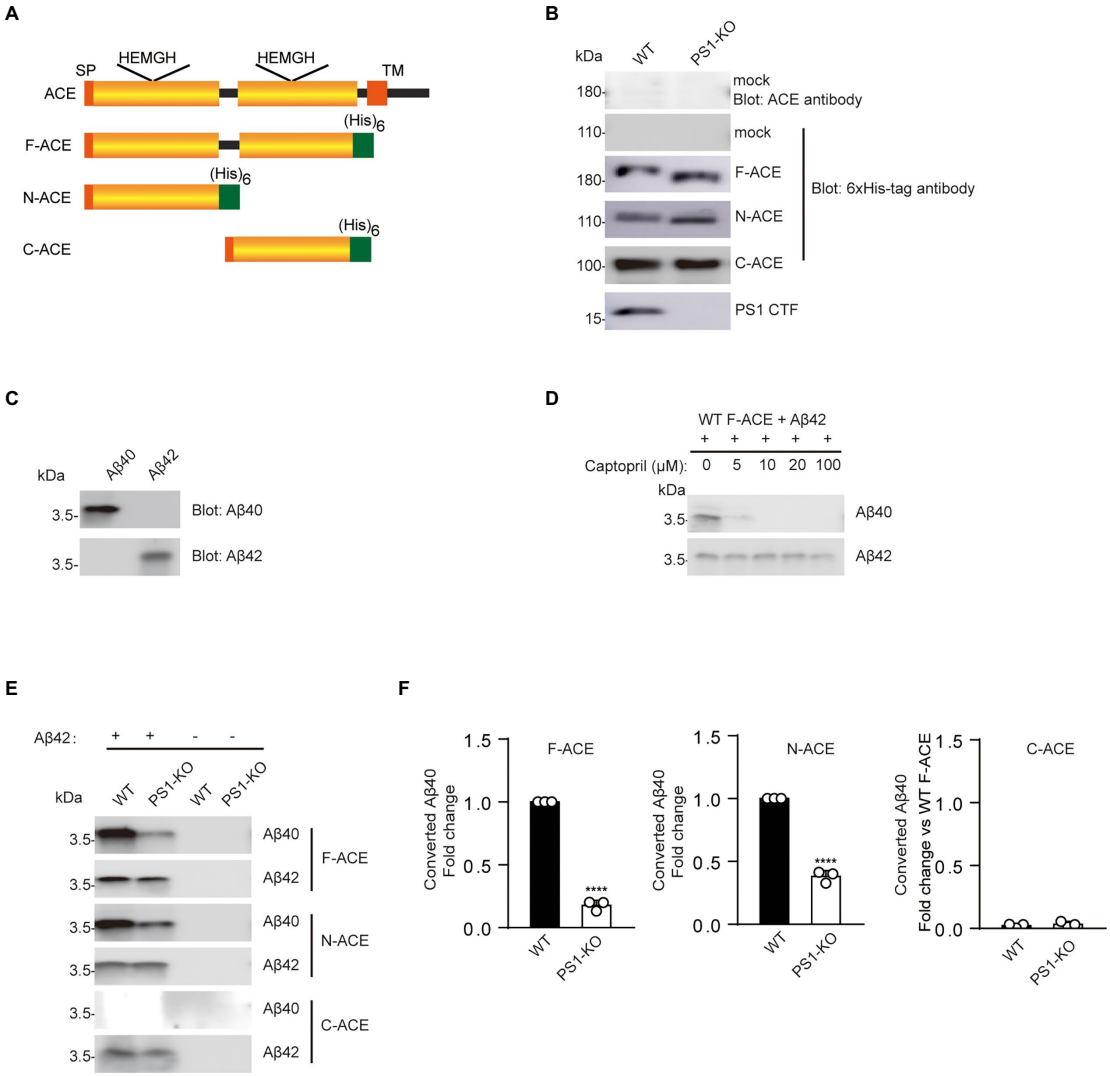
Recombinant F-ACE and N-ACE from PS1-KO fibroblasts exhibit decreased Aβ42-to-Aβ40-converting activity. **(A)** Schematic representation of human ACE and recombinant ACE proteins. The wild-type (WT) ACE protein consists of a signal peptide (SP), a single transmembrane domain (TM), and two homologous catalytic domains (yellow boxes). Recombinant ACE proteins (F-ACE, N-ACE and C-ACE) contain 6 histidine residues (green box) at the C-terminus and a SP at the N-terminus. **(B)** Western blots of 20 μg protein from the lysate of WT and PS1-KO fibroblasts transfected with empty vectors (mock) were probed with anti-ACE and anti–6 × His-tag antibodies (Upper two panels). Western blots of 20 μg of purified F-, N-, and C-ACE proteins from WT or PS1-KO fibroblasts were probed with anti–6 × His-tag or PS1-CTF antibodies (Lower four panels). **(C)** The specificity of the anti-Aβ40 and anti-Aβ42 antibodies was confirmed by Western blotting of 0.1 μg of synthetic Aβ40 and Aβ42. **(D)** Captopril 0, 5, 10, 20, 100 μM was incubated with purified F-ACE protein and synthetic Aβ42 for 2 h. **(E)** Purified F-, N-, and C-ACE proteins were mixed with synthetic Aβ42 and incubated at 37°C for 2 h. Western blots of the mixture were probed with anti-Aβ40 and anti-Aβ42 antibodies. The symbols (+) and (−) indicate with and without synthetic Aβ42. **(F)** Quantification of Aβ40 converted from Aβ42. F-and N-ACE purified from PS1-KO cells showed lower Aβ-converting activity (Aβ42-to-Aβ40–converting activity) compared with proteins from WT fibroblasts. C-ACE did not show any Aβ-converting activity. Values represent the mean ± SEM; *n* = 3; ****, *p* < 0.0001, by unpaired two-tailed Student’s *t* test. F-ACE, full-domain ACE; N-ACE, N-terminal domain ACE; C-ACE, C-terminal domain ACE.

To determine whether PS1 regulates ACE maturation and its Aβ42-to-Aβ40-converting activity, we purified three different recombinant ACE proteins from WT and PS1-KO fibroblasts. Full-domain ACE (F-ACE) includes both the N-terminal and C-terminal domain active sites. N-ACE includes only the N-terminal active site, whereas C-ACE includes only the C-terminal active site (Figure 1A; Zou et al., 2009). A 6-histidine tag was used to the C-terminus of all ACE mutants to facilitate purification. WT and PS1-KO fibroblasts transiently overexpressed F-ACE, N-ACE, and C-ACE. No endogenous ACE was detected in the lysate of fibroblasts transfected with empty vectors (mock) using anti-ACE and anti–6 × His-tag antibodies. The F-ACE, N-ACE, and C-ACE proteins were purified after overexpression in fibroblasts and then examined by Western blotting. The molecular weight of F-ACE and N-ACE from PS1-KO fibroblasts was slightly lower than that of the proteins from WT fibroblasts (Figure 1B), suggesting that PS1 deficiency affects the maturation or glycosylation of F-ACE and N-ACE. However, there was no difference in the molecular weight of C-ACE purified from WT and PS1-KO fibroblasts (Figure 1B).

As indicated above, ACE has two active domains, with the Aβ42-to-Aβ40-converting activity localized in the N-terminal domain and the angiotensin-converting activity localized in the C-terminal domain (Zou et al., 2009). Synthetic Aβ40 and Aβ42 were used to examine the specificity of the anti-Aβ40 and anti-Aβ42 antibodies, and no cross-reaction was observed (Figure 1C). To identify whether the generation of Aβ40 is dependent on Aβ42-to-Aβ40-converting activity of ACE, we incubated an ACE inhibitor captopril with the mixture of F-ACE and synthetic Aβ42. Aβ40 generation was completely inhibited by captopril (Figure 1D). To determine whether PS1 deficiency affects the Aβ42-to-Aβ40-converting activity of ACE, we incubated purified ACE proteins from WT or PS1-KO fibroblasts with Aβ42 and examined the generation of Aβ40 using anti-Aβ40 and anti-Aβ42 antibodies. As reported in our previous study, only the F-ACE and N-ACE domains exhibited Aβ42-to-Aβ40-converting activity (Figures 1E,F). Interestingly, F-ACE and N-ACE purified from PS1-KO cells showed significantly lower Aβ42-to-Aβ40-converting activity compared to the domains purified from WT cells (Figures 1E,F). These results suggest that PS1 deficiency affects the maturation/glycosylation of ACE and reduces the Aβ42-to-Aβ40-converting activity of F-ACE and N-ACE.

### PS1 deficiency completely abolished the angiotensin-converting activity of ACE

**Figure 2:**
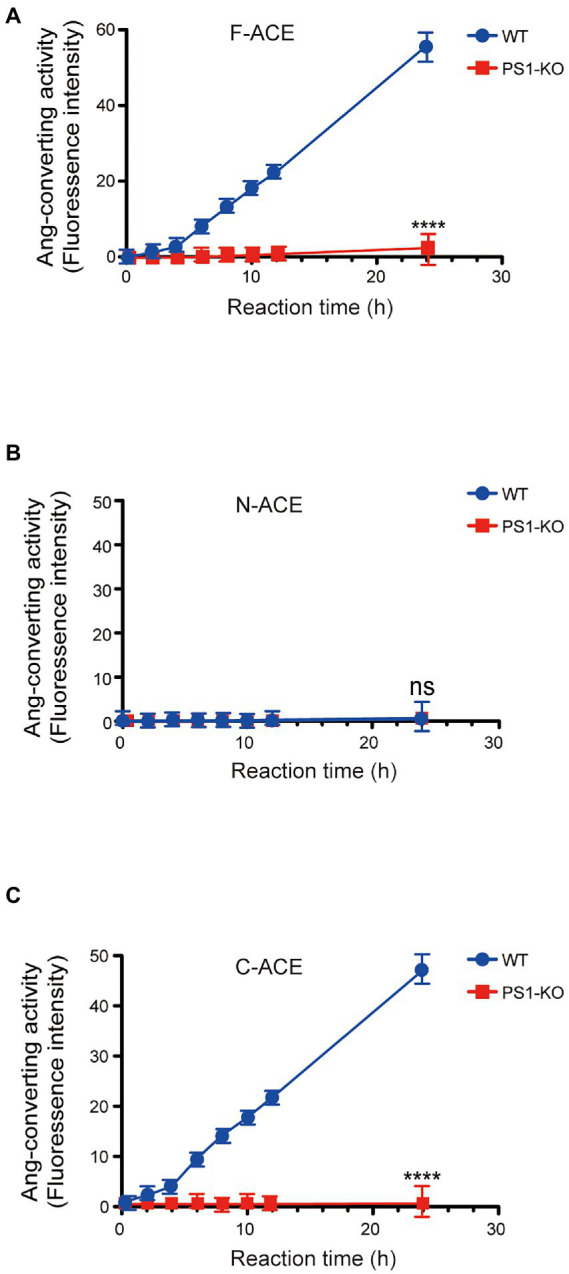
Loss of angiotensin-converting activity in recombinant F-and C-ACE from PS1-KO fibroblasts. Ang-converting (angiotensin-converting) activity was measured by incubating 2 μg of ACE protein with the synthetic o-aminobenzoyl peptide substrate for 24 h at 37°C. **(A)** F-ACE purified from WT fibroblasts showed Ang-converting activity, whereas PS1-KO fibroblasts did not show any Ang-converting activity. **(B)** N-ACE purified from WT and PS1-KO fibroblasts did not show any Ang-converting activity. **(C)** C-ACE purified from WT fibroblasts showed Ang-converting activity, whereas PS1-KO fibroblasts did not show any Ang-converting activity. Values represent the means ± SD; *n* = 3; ****p < 0.0001. NS, not significant, by unpaired two-tailed Student’s *t* test.

We also examined the angiotensin-converting activity of F-ACE, N-ACE, and C-ACE purified from WT and PS1-KO fibroblasts. The angiotensin-converting activity of these proteins was analyzed by monitoring the cleavage of a synthetic *o*-aminobenzoyl peptide substrate to release a fluorophore. As previously reported, F-ACE and C-ACE purified from WT fibroblasts exhibited angiotensin-converting activity, whereas N-ACE did not (Figures 2A–C). Surprisingly, the angiotensin-converting activity of F-ACE and C-ACE purified from PS1-KO cells was completely abolished (Figures 2A,C). These results suggest that PS1 is essential for the angiotensin-converting activity ACE.

### PSEN1 mutations reduce the Aβ42-to-Aβ40-converting activity of ACE

**Figure 3:**
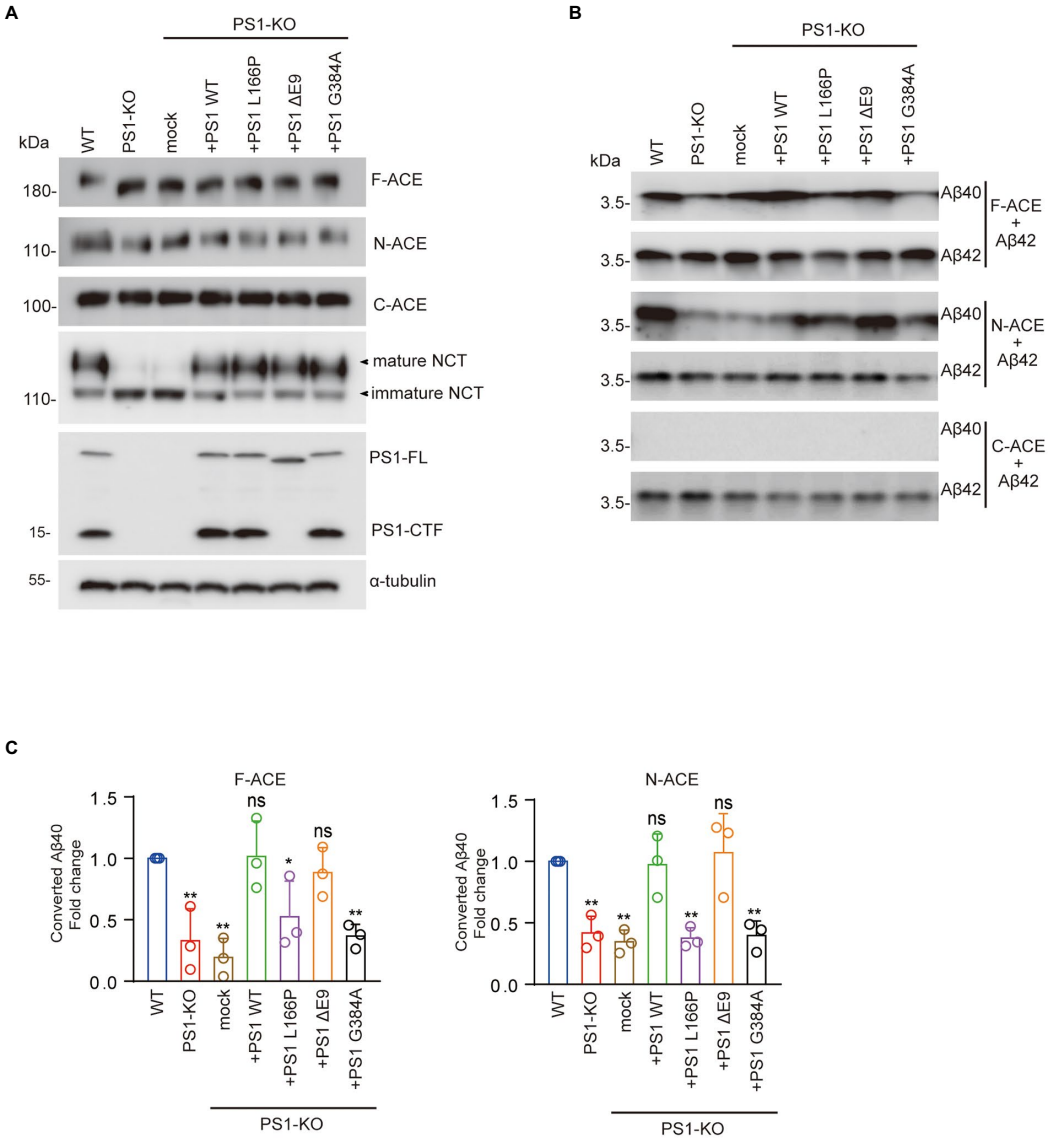
FAD-linked PS1L166P and PS1G384A mutations did not restore Aβ42-to-Aβ40–converting activity of ACE. **(A)** Fibroblasts were transfected with empty vector or PS1WT or PS1 mutant plasmids. Recombinant ACE proteins, NCT, PS1, and α-tubulin were detected by Western blotting. **(B)** F-, N-, and C-ACE purified from WT and PS1-KO fibroblasts or PS1-KO fibroblasts transfected with PS1WT or PS1 mutant were mixed with synthetic Aβ42 and incubated at 37°C for 2 h. Western blots of the mixtures were probed with anti-Aβ40 and anti-Aβ42 antibodies. **(C)** Quantification of Aβ40 converted from Aβ42. PS1L166P and PS1G384A did not restore the Aβ42-to-Aβ40–converting activity of F-ACE and N-ACE proteins, whereas PS WT and PS1ΔE9 restored this activity. Values represent the mean ± SD; *n* = 3; *, *p* < 0.05, **, *p* < 0.01. NS, not significant, Holm-Sidak’s multiple comparisons test.

To determine whether *PSEN1* mutations affect the Aβ42-to-Aβ40-converting activity of ACE, we transfected PS1WT, PS1L166P, PS1ΔE9, or PS1G384A into PS1-KO fibroblasts. We then transfected F-ACE, N-ACE, or C-ACE into these fibroblasts and purified the ACE proteins from the respective transfectants. Western blots of the purified ACE proteins are shown in Figure 3A. The PS1WT and PS1 mutants partially restored the maturation of the F-ACE and N-ACE proteins (Figure 3A). The level of mature NCT was significantly reduced in PS1-KO fibroblasts; however, NCT maturation was completed restored by transfection of the PS1WT and PS1 mutants (Figure 3A).

We then examined the Aβ42-to-Aβ40-converting activity of the ACE proteins by incubating them in the presence of Aβ42. Interestingly, PS1WT and PS1ΔE9 restored the Aβ42-to-Aβ40-converting activity of both F-ACE and N-ACE to levels similar to those of F-ACE and N-ACE from WT fibroblasts. However, PS1L166P and PS1G384A did not restore the Aβ42-to-Aβ40-converting activity of F-ACE and N-ACE compared with PS1WT (Figures 3B,C). As previously reported, none of the C-ACE proteins exhibited Aβ42-to-Aβ40-converting activity (Figures 3B,C). These results suggest that some *PSEN1* mutations increase the Aβ42/40 ratio by reducing the Aβ42-to-Aβ40-converting activity of ACE.

### PS1WT and PS1 mutants restored the angiotensin-converting activity of ACE in PS1-KO fibroblasts

**Figure 4:**
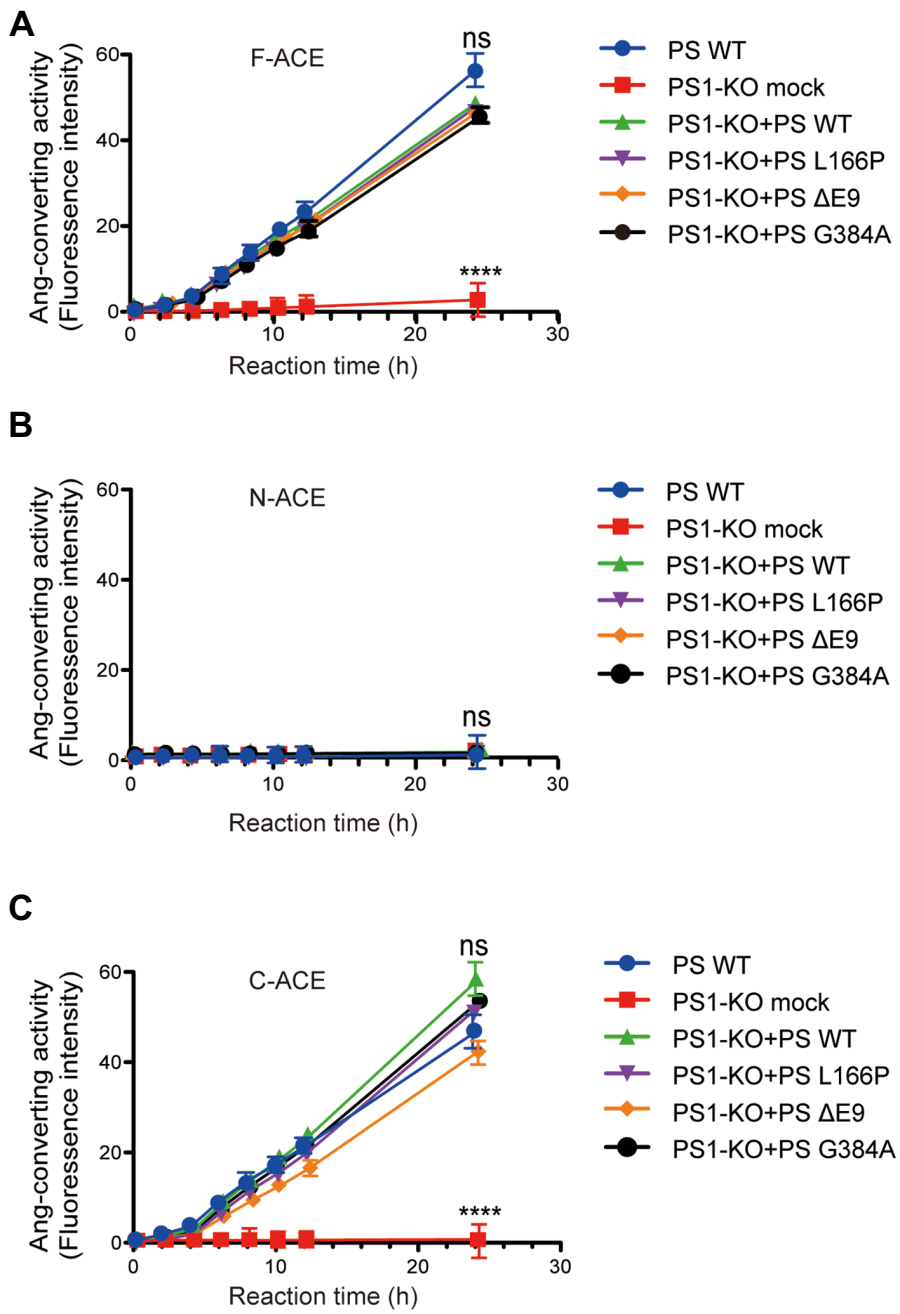
Transfection of PS1WT and PS1 mutants restored angiotensin-converting activity of F-ACE and C-ACE in PS1-KO fibroblasts. Ang-converting activity was measured by incubating 2 μg of ACE protein with the synthetic o-aminobenzoyl peptide substrate for 24 h at 37°C. **(A)** Transfection of PS1WT and PS1 mutants fully restored Ang-converting activity of F-ACE in PS1-KO fibroblasts. **(B)** N-ACE purified from WT, PS1-KO, and PS1-KO fibroblasts transfected with PS1WT and PS1 mutants did not show any ang-converting activity. **(C)** Transfection of PS1WT and PS1 mutants fully restored the Ang-converting activity of C-ACE in PS1-KO fibroblasts. PS1WT and PS1 mutants did not exert different effects on angiotensin-converting activity. Values represent the mean ± SD; *n* = 3; NS, not significant, ****p < 0.0001, Holm-Sidak’s multiple comparisons test.

We also examined the angiotensin-converting activity of F-ACE, N-ACE, and C-ACE proteins purified from PS1-KO fibroblasts transfected with PS1WT, PS1L166P, PS1ΔE9, or PS1G384A. In contrast to the Aβ42-to-Aβ40-converting activity, all of the PS1WT and PS1 mutants of F-ACE and C-ACE proteins exhibited angiotensin-converting activity (Figures 4A,C). Because angiotensin-converting activity is not localized in the N-terminal domain of ACE, this activity was not detected after transfection of PS WT and PS1 mutants (Figure 4B). These results suggest that PS1 is essential for the angiotensin-converting activity of ACE and that FAD-linked PS1 mutants do not affect the angiotensin-converting activity.

PS1 deficiency and PS1 mutant reduced the Golgi apparatus distribution of ACE.

**Figure 5:**
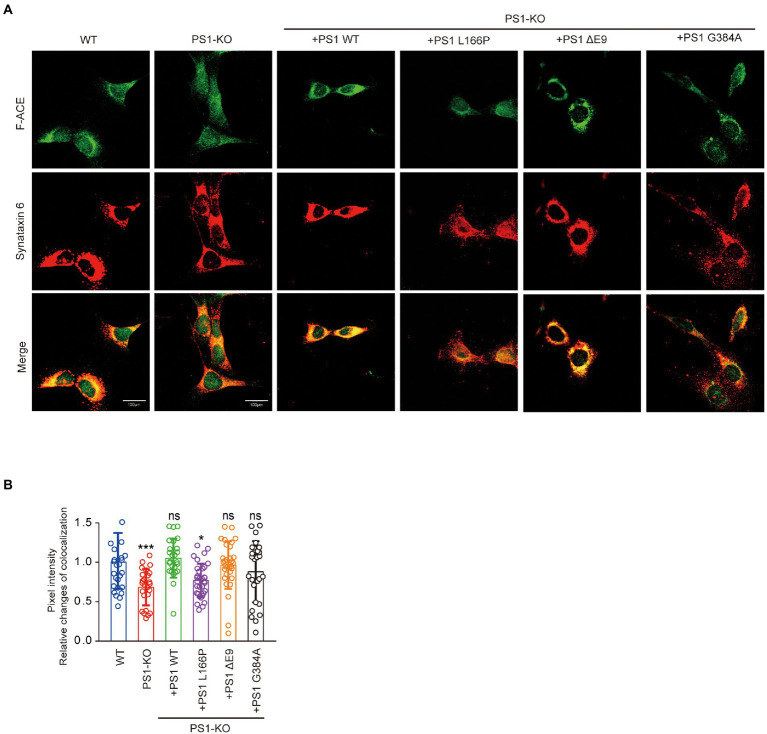
PS1 deficiency and PS1 mutant affected the localization of ACE. **(A)** Immunostaining for Golgi apparatus (red) and F-ACE (green) in WT, PS1-KO, and PS1-KO fibroblasts transfected with PS1WT and PS1 mutants. Scale bars, 100 μm. **(B)** Quantification of ACE intensity in Golgi apparatus of WT, PS1-KO, and PS1-KO fibroblasts transfected with PS1WT and PS1 mutants. *n* ≥ 25 different stained cells/group. *p < 0.05, ***p < 0.001. NS, not significant, Holm-Sidak’s multiple comparisons test.

To gain mechanistic insights into the decreased Aβ42-to-Aβ40-and angiotensin-converting activities of ACE protein in PS1-KO fibroblasts, we investigated whether the Golgi apparatus distribution of ACE changed in the cells. We found that the localization of F-ACE protein in Golgi apparatus decreased in PS1-KO fibroblasts. Transfection of PS1WT, PSΔE9 and PS1G384A into PS1-KO fibroblasts restored the distribution of F-ACE in Golgi apparatus, however, PS1L166P did not restored the Golgi apparatus distribution of F-ACE (Figures 5A,B). These results suggest that reduced distribution of ACE in Golgi apparatus can decrease ACE maturation and impair its activities.

De-glycosylation of ACE abolished its Aβ42-to-Aβ40-and angiotensin-converting activities.

**Figure 6:**
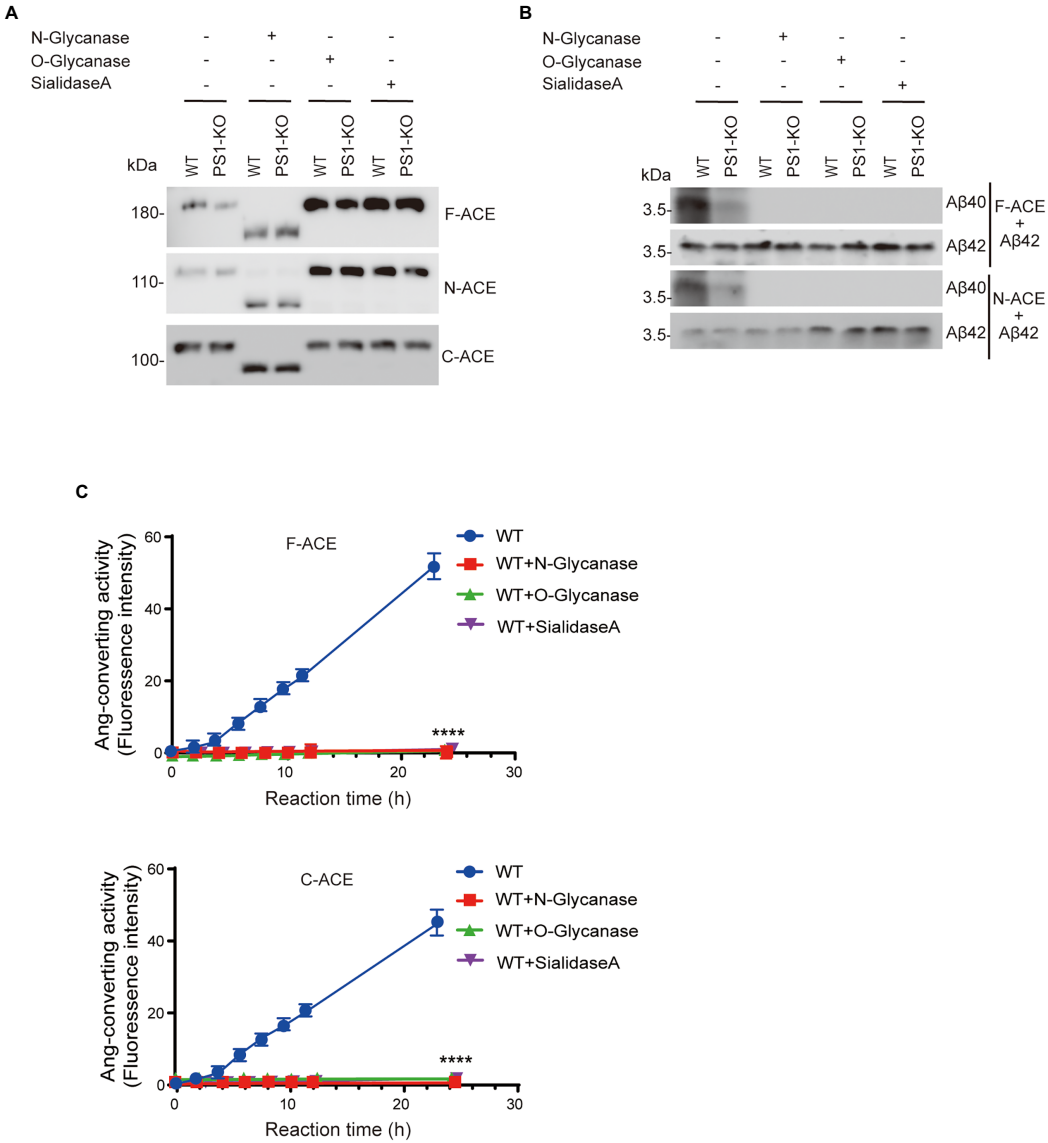
De-glycosylation abolished the Aβ42-to-Aβ40– and angiotensin-converting activity of ACE. **(A)** Purified F-ACE, N-ACE, and C-ACE (30 μg each) from WT and PS1-KO fibroblasts was de-glycosylated using 1 μl of *N*-glycanase, *O*-glycanase, or sialidase A for 2 h at 37°C. Western blots of 20 μg of de-glycosylated F-, N-, or C-ACE proteins from WT or PS1-KO fibroblasts were probed with an anti–6 × His-tag antibody. **(B)** De-glycosylated F-ACE and N-ACE proteins were mixed with synthetic Aβ42 and incubated at 37°C for 2 h. Western blots of the mixtures were probed with anti-Aβ40 and anti-Aβ42 antibodies. F-ACE and N-ACE did not exhibit Aβ-converting activity after de-glycosylation using *N*-glycanase, *O*-glycanase, or sialidase A. **(C)** F-ACE and C-ACE did not exhibit Ang-converting activity after de-glycosylation using *N*-glycanase, *O*-glycanase, or sialidase A. Values represent the mean ± SD; *n* = 3; ****, *p* < 0.0001, Holm-Sidak’s multiple comparisons test.

To examine which glycosylation is necessary for Aβ42-to-Aβ40-and angiotensin-converting activities, F-ACE, N-ACE, and C-ACE were incubated with *N*-glycanase, *O*-glycanase, or sialidase A. After treatment with *N*-glycanase, the molecular weight of F-ACE, N-ACE, and C-ACE was significantly reduced, indicating that most glycosylation of ACE is *N*-glycan. *O*-Glycanase and sialidase A treatment did not significantly reduce the molecular weight of the ACE proteins compared with *N*-glycanase (Figure 6A). We then incubated Aβ42 with the de-glycosylated F-ACE and N-ACE proteins. After de-glycosylation by *N*-glycanase, *O*-glycanase, or sialidase A, neither F-ACE nor N-ACE exhibited Aβ42-to-Aβ40-converting activity (Figure 6B). Similarly, the angiotensin-converting activity of F-ACE and C-ACE was also abolished by treatment with *N*-glycanase, *O*-glycanase, or sialidase A (Figure 6C).

Aβ42-to-Aβ40-converting activity was lower in adult mouse brain cortex than embryonic brain cortex.

**Figure 7:**
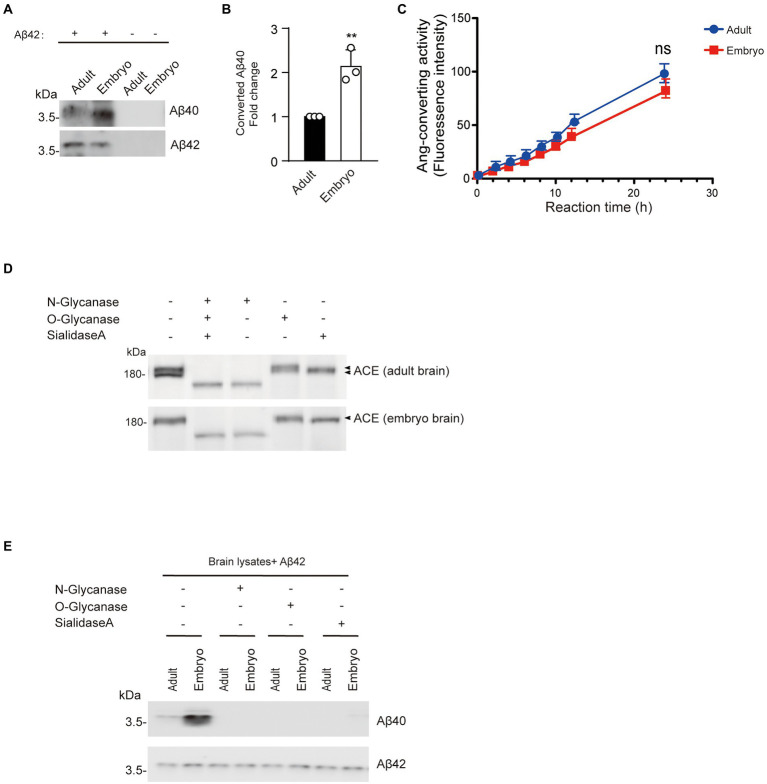
Aβ42-to-Aβ40-converting activity in adult mouse brain cortex is lower than that in embryonic brain cortex. **(A)** Synthetic Aβ42 protein (40 μM) was incubated with 20 μg of cortex lysate from 17-day-old embryos or 3-month-old mice. Aβ40 and Aβ42 were detected by Western blotting. **(B)** Quantification of Aβ40 converted from Aβ42. Embryo cortex exhibited higher Aβ42-to-Aβ40-converting activity than adult cortex. Values represent the mean ± SEM; *n* = 3; **p < 0.001, by unpaired two-tailed Student’s *t* test. **(C)** Ang-converting activity was measured by incubating 2 μg of total protein of cortex lysate from 17-day-old embryos or 3-month-old mice with the synthetic o-aminobenzoyl peptide substrate for 24 h at 37°C. Values represent the mean ± SD; *n* = 3; NS, not significant, by unpaired two-tailed Student’s *t* test. **(D)** Western blots of 20 μg of total protein of cortex lysate from 17-day-old embryos or 3-month-old mice were probed with a polyclonal anti-ACE antibody. **(E)** 20 μg protein of cortex lysate from 17-day-old embryos or 3-month-old mice were de-glycosylated and incubated with synthetic Aβ42. Aβ40 and Aβ42 were detected by Western blotting.

To determine whether changes in brain Aβ42-to-Aβ40-converting activity are development dependent, we incubated cortex lysate from 17-day-old embryos or 3-month-old mice with synthetic Aβ42. The level of Aβ40 converted from Aβ42 in adult cortex was lower than that in embryonic cortex, indicating that embryonic cortex has higher Aβ42-to-Aβ40-converting activity than adult cortex (Figures 7A,B). However, there was no difference in angiotensin-converting activity between the cortex lysates from 17-day-old embryos and 3-month-old mice (Figure 7C). Interestingly, ACE protein in adult cortex showed two bands on Western blotting, whereas a single band corresponding to the upper band of ACE in adult brain was observed in the embryonic cortex (Figure 7D). After de-glycosylation with *N*-glycanase, the molecular weight of both adult and embryonic brain ACE decreased to a single band of approximately 150 kDa, whereas *O*-glycanase did not significantly change the molecular weight of ACE. Notably, sialidase A slightly reduced the molecular weight of the upper band of ACE from adult brain and ACE from embryonic brain (Figure 7D). Then we also examined the Aβ42-to-Aβ40-converting activity in the brain lysate after de-glycosylation. Aβ40 generation was not detected after de-glycosylation with *N*-glycanase, *O*-glycanase, or sialidase A (Figure 7E). These results suggest that the Aβ42-to-Aβ40-converting activity of ACE decreases with development in adult brain compared with embryonic brain and that glycosylation modulates the Aβ42-to-Aβ40-converting activity of ACE.

## Materials and methods

### Cell culture and transfection

WT and PS1-KO mouse embryonic fibroblasts (MEFs) were maintained in Dulbecco’s modified Eagle’s medium (Wako, Osaka, Japan) containing 10% fetal bovine serum (Corning, Woodland, CA) at 37°C in a 5% CO_2_ atmosphere. To overexpress human ACE (F-ACE, N-ACE, and C-ACE with 6 × His-tag) and the PS1WT and PS mutants (PS1L166P, PS1ΔE9, and PS1G384A), plasmids encoding the target cDNAs were transfected into platinum-E cells using FuGENE (Promega, Madison, WI, United States) for packaging. Conditioned medium was collected after 48 h and added to MEFs. In order to increase the transfection efficiency, polybrene was added at the same time to a final concentration of 5 μg/ml.

### ACE protein purification

Cells transfected with F-ACE, N-ACE, and C-ACE were harvested in lysis buffer [50 mM Tris–HCl (pH 7.5) containing 0.5% NP40] and centrifuged at 15,000 *g* for 30 min. Next, 1 ml of TALON^®^ metal affinity resin (Takara, Shiga, Japan) was used to purify ACE protein from 8 ml of MEF lysate. All of the abovementioned operations were carried out at 4°C. The concentration of purified ACE protein was determined using a Pierce™ BCA protein assay kit (Thermo Scientific, Rockford, IL, United States).

### Mouse cortex sample

WT C57BL/6 J female mice at gestation day 17 were perfused with PBS (137 mM NaCl, 2.7 mM KCl, 10 mM Na_2_HPO_4_, 1.8 mM KH_2_PO_4_, 1 mM CaCl_2_, and 0.5 mM MgCl_2_). The cortex from 3-month-old mice and embryos was homogenized with lysis buffer [50 mM Tris–HCl (pH 7.5) containing 0.5% NP40] and centrifuged at 15000 *g* and 4°C for 30 min. The protein concentration of the brain lysate was determined using a Pierce™ BCA Protein assay kit (Thermo Scientific). The experiments in this study were performed in strict accordance with the recommendations of the Fundamental Guidelines for Proper Conduct of Animal Experiments and Related Activities in Academic Research Institutions, under the jurisdiction of the Ministry of Education, Culture, Sports, Science and Technology, Japan.

### Western blot

A total of 5 μg of cell lysate protein or ACE and 20 μg of protein from adult and embryonic mouse cortex were separated by SDS-PAGE and transferred onto PVDF membranes (Sigma-Aldrich, St. Louis, MO, United States). The membranes were incubated overnight at 4°C with the proper primary antibodies. After incubating with appropriate peroxidase-conjugated secondary antibodies, the membranes were visualized using Super Signal Chemiluminescence (Wako) and an Amersham Imager 680. Detailed information regarding antibodies is provided in Supplementary Table S1.

### Aβ42-to-Aβ40-converting activity assay

Purified ACE protein (25 μg) was mixed with freshly dissolved synthetic Aβ42 (PEPTIDE, Osaka, Japan) at a final concentration of 40 μM (Zou et al., 2009). The mixture was incubated at 37°C for 4 h and then dissolved in 2× SDS sample buffer [0.125 M Tris–HCl (pH 6.8), 20% glycerol, 4% SDS, 10% 2-mercaptoethanol, 0.004% bromophenol blue]. The mixture was separated by SDS-PAGE in tricine buffer and blotted onto a PVDF membrane. Aβ40 and Aβ42 were detected using anti-Aβ40 and anti-Aβ42 antibodies.

### Angiotensin-converting activity assay

ACE activity was analyzed using an ACE activity assay kit (abcam, Cambridge, United Kingdom) according to the manufacturer’s instructions. A total of 2 μg of ACE protein was mixed with a synthetic o-aminobenzoyl peptide substrate and incubated at 37°C. ACE activity was measured every 2 h using a fluorescence microplate reader at Ex/Em wavelengths of 330/340 nm.

### ACE de-glycosylation

An ACE de-glycosylation kit (abcam) was used to de-glycosylate purified ACE protein according to the manufacturer’s instructions. A total of 30 μg of ACE protein was mixed with PNGase F, *O*-glycosidase, or α-2(3, 6, 8, 9)-neuraminidase to remove *N*-glycosylation, *O*-glycosylation, or sialic acid. De-glycosylated ACE was analyzed by Western botting, and its activity was examined.

### Immunofluorescence staining

WT fibroblasts, PS1-KO fibroblasts transfected with F-ACE, and PS1-KO fibroblasts transfected with F-ACE and PS1 mutants were seeded using image culture dishes (Eppendorf) and incubated at 37°C for 24 h. We firstly fixed cells in 4% paraformaldehyde for 30 min at room temperature. The cells were then permeabilized with 0.1% Triton X-100 for 20 min and incubated in 10% donkey serum in Tris-buffered saline containing 0.05% Tween 20 for 1 h at room temperature. The cells were incubated overnight at 4°C with anti-syntaxin-6 and anti-ACE antibodies. Immunofluorescent labeling by staining with Alexa Fluor 488 or Alexa Fluor 568-conjugated secondary antibodies. Images were acquired with a confocal microscope (Olympus FV3000, Tokyo, Japan). Details on the antibodies used are provided in Supplementary Table S1. ImageJ software was used to quantify the colocalization of ACE with the Golgi apparatus. The threshold intensity for both fluorescent signals is preset, which is determined using a colocalization threshold function. Colocalized pixels above a threshold intensity were automatically quantified and scored, and results were expressed as colocalized mean intensity positivity for both channels. Supplementary Table S1 provides details on antibodies and reagents.

### Statistical analyzes

Prism 7.0 software (GraphPad Software, San Diego, CA) was used for statistical analyzes. All data are shown as the mean ± SEM or mean ± SD of at least three independent experiments, with *p* < 0.05 considered statistically significant. Student’s *t* tests were used to determine the significance of differences between two groups. Group differences were analyzed by one-way analysis of variance followed by ANOVA with Holm-Sidak’s multiple comparisons tests for multiple groups against the control group. All experiments produced similar results under the same or similar conditions, and normal distribution of the data was assumed.

## Discussion

PS1-KO mice developed obvious developmental defects in the embryonic period and eventually died in the perinatal period (Donoviel et al., 1999). Presenilin gene mutations account for the majority of FAD cases. Most FAD-related mutations in PS1 are associated with increased Aβ42 levels or decreased Aβ40 levels, which results in an elevated Aβ42/40 ratio due to loss of PS1 function (Fernandez et al., 2014). These data also suggest that declines in neuroprotective Aβ40 levels may contribute to the pathogenesis of AD (Zou and Michikawa, 2008). However, how *PSEN1* mutations lead to an increase in the Aβ42/40 ratio is unclear. Here, we examined whether PS1 can regulate the activity of ACE, which converts neurotoxic Aβ42 to neuroprotective Aβ40. For the first time, we demonstrated that PS1 deficiency leads to significant lower Aβ42-to-Aβ40-converting activity of ACE (Figure 1). Strikingly, ACE purified from PS1-KO fibroblasts did not show any angiotensin-converting activity (Figure 2). These results suggest that PS plays a crucial role in ACE maturation and activity, and also in blood pressure regulation.

Overexpression of WT PS1 in PS1-KO fibroblasts restored the Aβ42-to-Aβ40-and angiotensin-converting activities of ACE. Interestingly, some PS1 mutants successfully restored the angiotensin-converting activity of ACE but not its Aβ42-to-Aβ40-converting activity, suggesting that *PSEN1* mutations increase the Aβ42/40 ratio by impairing the Aβ42-to-Aβ40-converting activity of ACE. A previous study found that Aβ40 levels are reduced to <5% of PS1WT levels in PS1/2-KO fibroblasts as a result of the FAD-linked *PSEN1* mutations L166P and G384A, whereas PS1ΔE9 fibroblasts exhibit higher Aβ40 levels than PS1L166P and PS1G384A fibroblasts (Heilig et al., 2013). Patients with PS1L166P, PS1G384A, and PS1ΔE9 exhibit mean AD onset at 24 years, 35 years, and 45.5 years, respectively (Julliams et al., 1999; Cacquevel et al., 2012). These results suggest that lower Aβ40 levels are associated with earlier FAD onset. NCT undergoes a typical ER-to-Golgi maturation pattern, with most mature species localized to the Golgi (Yang et al., 2002). Multiple studies have shown that complex glycosylation of NCT is dependent on PS1, with strong downregulation of mature NCT levels observed in cells lacking PS1 (Figure 3A; Edbauer et al., 2002; Herreman et al., 2003). In our study, PS1WT, PS1ΔE9, PS1L166P, and PS1G384A completely restored the maturation of NCT and angiotensin-converting activity (Figures 3A, 4). However, PS1L166P did not restore the localization of F-ACE protein localized in Golgi apparatus (Figures 5A,B). In addition, a study showed that overexpression of PS1 with a familial AD mutation (M146L) in the neuroblastoma cell line SH-SY5Y resulted in reduced sialylation of NCAM (Farquhar et al., 2003). Thus, PS1 mutants may also reduce sialylation of ACE in Golgi apparatus. The detail structure of ACE glycan in PS1 mutant cells need to be further analyzed. In contrast to PS1L166P and PS1G384A, only PS PS1ΔE9 restored the Aβ42-to-Aβ40-converting activity of ACE (Figure 3). Thus, our results suggest that the PS1L166P and PS1G384A mutations result in low Aβ42-to-Aβ40-converting activity, which leads to low Aβ40 levels and early onset of FAD.

Mammalian somatic ACEs contain two homology domains, an N-terminal domain (N-domain) and a C-terminal domain (C-domain), each with a zinc-dependent active site (Hooper and Turner, 2003). The presence of two active sites in ACE has inspired many attempts to determine whether the active sites differ functionally. ACE also hydrolyzes multiple polypeptide substrates, including substance P, luteinizing hormone–releasing hormone, acetyl-Ser-Asp-Lys-Pro (AcSDKP), and neurotensin (Turner and Hooper, 2002). AcSDKP, a peptide thought to inhibit myeloid maturation, is preferentially cleaved by the N-domain of ACE *in vitro* (Rousseau et al., 1995). In contrast, the ACE C-domain is the major site of angiotensin I cleavage *in vivo* (Fuchs et al., 2008).

Similar to our previous report, here, we also found that the angiotensin-converting activity of ACE is localized in the C-domain, whereas the Aβ42-to-Aβ40-converting activity is specifically localized in the N-domain (Figures 1E, 2B). We previously purified overexpressed F-ACE, N-ACE, and C-ACE proteins from cell culture medium (Zou et al., 2009). However, PS1-KO fibroblasts did not secrete any ACE protein after overexpression, possibly because PS1 deficiency impairs cellular secretion (Islam et al., 2022). Thus, we purified ACE protein from cell lysate, and this ACE protein exhibited activity similar to that of ACE purified from culture medium (Figures 1, 2).

Somatic ACE is highly glycosylated, and its glycan structure, as well as the location of the oligosaccharide chains, can vary with different protein sources (Kryukova et al., 2015). The sequence of human somatic ACE includes 17 potential *N*-glycosylation sites and 2 *O*-glycosylation sites (Anthony et al., 2010; Goth et al., 2015). We previously found that *N*-glycosylation is required for the Aβ42-to-Aβ40-and angiotensin-converting activities of ACE. Here, we found that removal of either *N*-glycosylation, *O*-glycosylation, or sialic acid abolished the Aβ42-to-Aβ40-and angiotensin-converting activities of ACE (Figures 4B,C). Our results suggest that the presence of *N*-glycosylation, *O*-glycosylation, or sialic acid plays an essential role in the Aβ42-to-Aβ40-and angiotensin-converting activities of ACE. NCT maturation has been reported to be reduced during rat brain development (Uchihara et al., 2006). We also found that glycosylation and Aβ42-to-Aβ40-converting activity of ACE decreases with development in adult brain compared with embryonic brain (Figures 7A,B).

**Figure 8:**
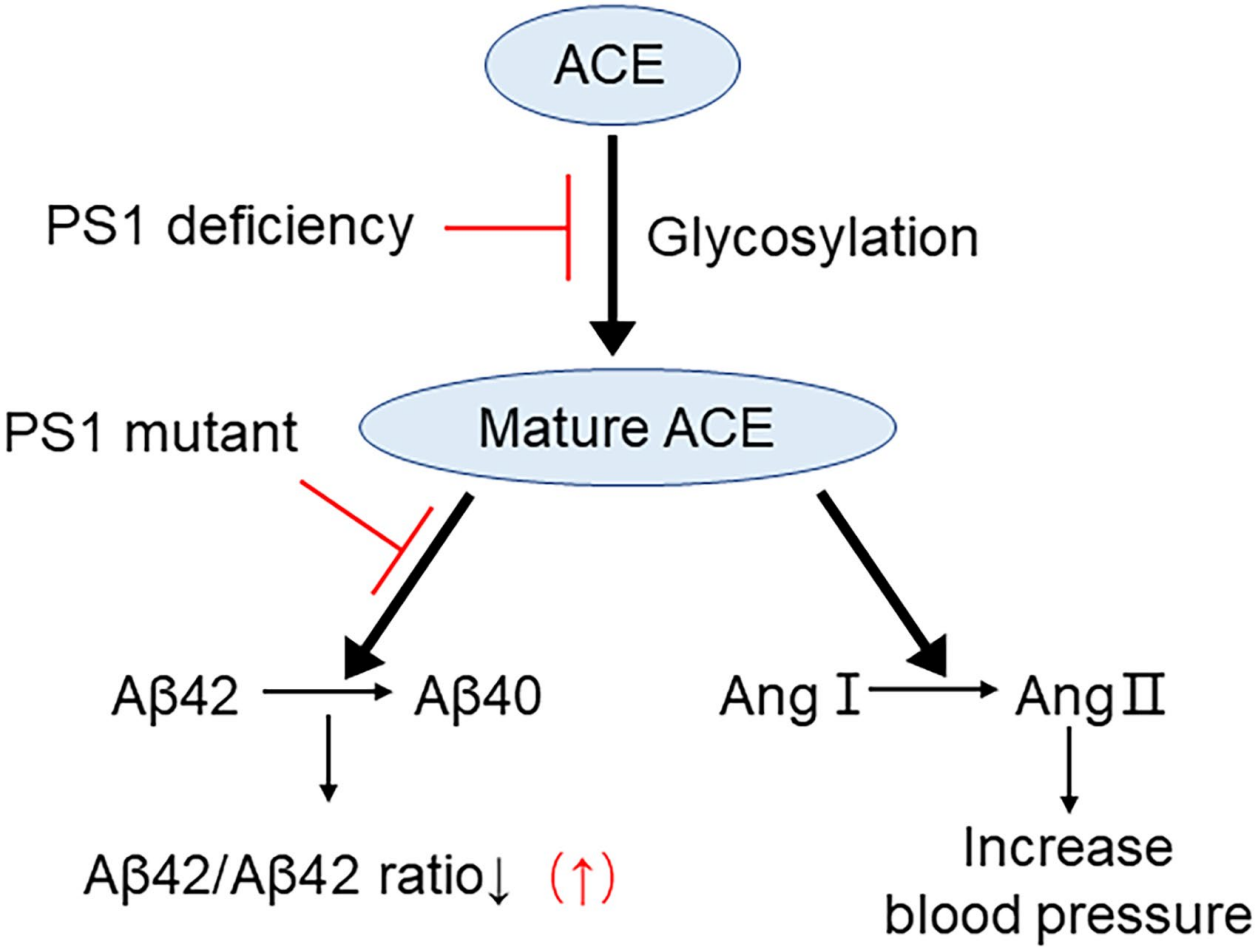
Proposed mechanism on PS1 mutant elevating Aβ42/40 ratio by impairing ACE maturation. ACE maturation/glycosylation is essential for its Aβ42-to-Aβ40-and angiotensin-converting activities and for reducing Aβ42/40 ratio. PS1 deficiency inhibits ACE maturation and both of these activities. PS1 mutant selectively inhibits Aβ42-to-Aβ40-converting activity of ACE and elevates Aβ42/40 ratio. However, PS1 mutants do not inhibit angiotensin converting activity of ACE.

Collectively, our data indicate that deletion of PS1 results in a significant decrease in both the Aβ42-to-Aβ40-converting activity and angiotensin-converting activity of ACE. Moreover, some FAD-linked *PSEN1* mutations were shown to impair the Aβ42-to-Aβ40-converting activity of ACE (Figure 8). Our results suggest that the increase in the Aβ42/40 ratio associated with FAD-linked *PSEN1* mutations results from not only altered γ-secretase cleavage but also the decrease in the Aβ42-to-Aβ40-converting activity of ACE. In addition, the presence of the *ACE I* allele with decreased serum and tissue ACE levels appears to be strongly associated with AD onset (Lehmann et al., 2005). Thus, approaches that maintain or enhance the Aβ42-to-Aβ40-converting activity of ACE will be useful for reducing the Aβ42/40 ratio and preventing the onset of AD. Taken together, our results suggest that enhancing PS-mediated trafficking and maturation of ACE may decrease Aβ42/40 ratio and can be used as a strategy for developing novel therapeutic regimens for AD patients.

## Data availability statement

The original contributions presented in the study are included in the article/Supplementary material, further inquiries can be directed to the corresponding authors.

## Ethics statement

The animal study was reviewed and approved by The experiments in this study were performed in strict accordance with the recommendations of the Fundamental Guidelines for Proper Conduct of Animal Experiments and Related Activities in Academic Research Institutions, under the jurisdiction of the Ministry of Education, Culture, Sports, Science and Technology, Japan.

## Author contributions

YG, YS, TN, TT, and KZ: data curation. YG, YS, SI, and KZ: formal analysis. YG, YS, and KZ: investigation. YG and KZ: writing—original draft. YG, KZ, and MM: writing—review and editing. KZ: conceptualization and supervision. KZ and MM: funding acquisition and project administration.

## Funding

This work was supported by the Grant-in-Aid for Scientific Research C 19K07846 and 22K07352 (to KZ) from the Ministry of Education, Culture, Sports, Science, and Technology, Japan. This work was also supported by AMED under grant number JP20dk0207050h0001, JP20dk0207050h0002, JP20dk0207050h0003 and JP20de010702 (to MM), and by “the 24th General Assembly of the Japanese Association of Medical Sciences” (to KZ), the Daiko Foundation (to KZ), the Hirose International Scholarship Foundation (to KZ) and the Hori Sciences and Arts Foundation (to KZ).

## Conflict of interest

The authors declare that the research was conducted in the absence of any commercial or financial relationships that could be construed as a potential conflict of interest.

## Publisher’s note

All claims expressed in this article are solely those of the authors and do not necessarily represent those of their affiliated organizations, or those of the publisher, the editors and the reviewers. Any product that may be evaluated in this article, or claim that may be made by its manufacturer, is not guaranteed or endorsed by the publisher.

## Abbreviations

AD: Alzheimer’s disease
Aβ: amyloid β-protein
Aβ42: amyloid β-protein1-42
ACE: angiotensin-converting enzyme
PS: presenilin
PS1-CTF: C-terminal fragment of presenilin 1
NCT: nicastrin
APP: amyloid precursor protein
APH-1: anterior pharynx-defective-1
PEN-2: presenilin enhancer-2
FAD: Transmembrane domains
