# Supplementary material for: Presenilin 1 deficiency impairs Aβ42-to-Aβ40- and angiotensin-converting activities of ACE
Source: Front Aging Neurosci. 2023 Feb 17;15:1098034. doi: 10.3389/fnagi.2023.1098034 (PMC9981673; doi:10.3389/fnagi.2023.1098034)
Supplement: Supplementary file 1 [file Table_1.DOCX]

Table 1. Information regarding antibodies, reagents, and assay kits.

| REAGENT or RESOURCE | SOURCE | IDENTIFIER |
| --- | --- | --- |
| Primary antibodies |  |  |
| Mouse Anti-PS1 CTF | Merck Millipore | Catalog # MAB5232 |
| Rabbit Anti-NCT | Sigma-Aldrich | Catalog # N1660 |
| Mouse Anti-6xHis-tag | Thermofisher | Catalog #MA1-135 |
| Goat Anti-ACE | R&D | Catalog #AF1513 |
| Mouse Anti- Aβ40 | IBL | Catalog # 10047 |
| Rabbit Anti- Aβ42 | IBL | Catalog # 18582 |
| Anti-α-tubulin | Sigma-Aldrich | Catalog # T9026 |
| Mouse Anti-syntaxin 6 | BD Biosciences | Catalog # 610636 |
| Secondary antibodies |  |  |
| Anti-Mouse IgG HRP-linked Antibody | Cell Signaling | Catalog # 05/2019 |
| Anti-Rabbit IgG HRP-linked Antibody | Cell Signaling | Catalog # 09/2019 |
| Anti-Goat IgG HRP-linked Antibody | Merck Millipore | Catalog # AP186P |
| Alexa Fluor^TM^ 488 donkey anti-mouse IgG (H+L) | Thermofisher | Catalog # A-21202 |
| Alexa Fluor^TM^ 568 donkey anti-goat IgG (H+L) | Thermofisher | Catalog # A-11057 |
| Chemicals, antibiotics and recombinant proteins |  |  |
| Captopril | Wako | Catalog # 037-25273 |
| Polybrene | Santa Cruz Biotechnology | Catalog # sc-134220 |
| Puromycin dihydrochloride | Santa Cruz Biotechnology | Catalog # sc-108071 |
| FuGENE^®^ HD transfection reagent | Promega | Catalog # E231A |
| Enzymatic deglycosylation kit | Agilent Techologies | Catalog # GK80110 |
| Amyloid β-protein (Human, 1-42) | Peptide | Catalog #4349-v |
| ACE assay kit | abcam | Catalog #ab239703 |
